# Solid Food Introduction and the Development of Food Allergies

**DOI:** 10.3390/nu10111790

**Published:** 2018-11-17

**Authors:** Carlo Caffarelli, Dora Di Mauro, Carla Mastrorilli, Paolo Bottau, Francesca Cipriani, Giampaolo Ricci

**Affiliations:** 1 Clinica Pediatrica Unit, Department of Medicine and Surgery, University of Parma, 43126 Parma, Italy; dora.dimauro@hotmail.com (D.D.M.); carla.mastrorilli@icloud.com (C.M.); 2 Pediatria, Azienda Ospedaliera di Imola, Via Montericco 4, 40026 Imola, Italy; paolo.bottau@gmail.com; 3 Pediatric Unit, Department of Medical and Surgical Sciences, University of Bologna, Via Massarenti 11, 40138 Bologna, Italy; francy.cipriani@gmail.com (F.C.); giampaolo.ricci@unibo.it (G.R.)

**Keywords:** allergenic foods, complementary food, food allergy, egg allergy, peanut allergy, weaning, skin prick test, infants, IgE

## Abstract

The rise of food allergy in childhood, particularly among developed countries, has a significant weight on public health and involves serious implications for patients’ quality of life. Even if the mechanisms of food tolerance and the complex interactions between the immune system and environmental factors are still mainly unknown, pediatricians have worldwide implemented preventive measures against allergic diseases. In the last few decades, the prevention of food allergy has tracked various strategies of complementary feeding with a modification of international guidelines from delayed introduction to early weaning. Current evidence shows that complementary foods, including allergenic ones, should be introduced into diet after four months, or even better, following World Health Organization advice, around six months irrespective of risk for allergy of the individual. The introduction of peanut is recommended before 12 months of age among infants affected by severe eczema and/or egg allergy to diminish the occurrence of peanut allergy in countries with high peanut consumption. The introduction of heated egg at 6–8 months of age may reduce egg allergy. Infants at high risk of allergy similarly to healthy children should introduce complementary foods taking into account family and cultural preferences.

## 1. Introduction

In the 1990s, the strategies to prevent the development of food allergies (FA) and reduce the severity of eczema focused on the avoidance of food allergens in infancy. They were based on studies that identified the immaturity of the intestinal immune system as the main reason for failure of tolerance acquisition. As a result, international guidelines [1,2,3] recommended the avoidance of allergenic foods in pregnancy and during breastfeeding to prevent the onset of allergic diseases in infants with atopy in first*-*degree family members (high-risk infants). Additionally, the avoidance of allergenic complementary foods until the age of 12 months, eggs until 2 years and peanuts, tree nuts and fish until 3 years was recommended [1,2]. Despite measures of food avoidance, the incidence of FA in childhood continued to increase in Western countries [4,5,6], with serious consequences on patients’ and families’ quality of life and rising costs for society [7]. Furthermore, several studies have found that delayed exposure to allergenic foods did not reduce the risk of FA, questioning, however, if it could be responsible for its increasing frequency [8]. Consequently, updated guidelines [9,10,11,12,13] changed previous recommendations and recognized that there was no evidence that a delayed introduction of solid foods beyond 4–6 months was useful for the primary prevention of FA.

The unsatisfactory results of previous approaches led investigators to put forward new hypotheses to prevent food allergy (FA) [8]. One possibility is that allergic sensitization could occur through other routes of exposure and not only as a result of oral intake. This concept is supported by murine studies and observational human surveys, which show that skin exposure to food allergens is associated with allergic sensitization, especially in the case of barrier defects, as in children with atopic dermatitis and filaggrin deficiency [14,15,16,17]. Furthermore, epidemiological data found a significant increase in the prevalence of peanut sensitization in countries with high environmental exposure to peanut and late introduction of peanut into the infant’s diet [18]. On the other hand, the early introduction of some food allergens was associated with a reduction of FA. It was found that oral intake of ovalbumin (OVA) contributes to the development of OVA tolerance through induction of regulatory T cells in the small intestine [19]. The “dual-allergen exposure hypothesis” was therefore proposed, according to which early oral consumption of food allergens could induce oral tolerance, while allergic sensitization to food allergens could take place through the transcutaneous route [15,18,20,21,22].

The aim of this article is to summarize randomized studies that have investigated whether there is a time window for the early introduction of allergenic foods (both in infants at high-risk for atopy and in the general population) that could effectively prevent FA, ascertained by the diagnostic oral food challenge test [23]. Randomized trials were reported, since they provide a higher level of evidence to answer clinical questions. Information was derived from a literature search of the Pub-Med database for English-language randomized studies, meta-analyses and systematic reviews published in peer-reviewed journals. A previous systematic review [24] that found no randomized study on the preventive effect of early weaning was published before 30 September 2012. Therefore, in order to reflect new findings on the research topic for which the review was conducted, we restricted our search to studies conducted in the last 10 years. Other clinical trials, observational studies, case reports and conference abstracts were excluded from the study. The database search pattern used the following keywords: solid food introduction children atopy; complementary feeding; allergenic foods; peanut allergy; egg allergy; immunoglobulin E (IgE)-mediated; food allergy; and atopic dermatitis. Earlier relevant articles were identified by references used in retrieved sources or those already known to the authors. In the Conclusions, we provide a practical approach for the introduction of complementary foods to prevent FA, based on evidence from identified original articles, select reviews and existing recommendations, strengthened by the clinical experience of the authors to guide interpretation.

## 2. Timing of Introduction of Complementary Foods and Tolerance of Multiple Foods

### Introduction from 3–6 Months

The Enquiring About Tolerance (EAT) study [25] assessed whether the introduction of six common allergenic foods (peanuts, milk, egg, wheat, fish and sesame) could prevent FA in a general population of 1303 breast-fed infants at three months of age (Table 1). According to the intention-to-treat (ITT) analysis, 5.6% of infants with early introduction to foods between three and six months of life (early-introduction group (EIG)) developed FA to at least one of the six foods at three years versus 7.1% of infants with standard introduction (standard-introduction group (SIG)) of foods after six months. However, this difference was not significant for egg allergy and peanut allergy. The per-protocol (PP) analysis found a significant reduction not only of FA in EIG in comparison with SIG (2.4% vs. 6.4%), but also of peanut allergy and egg allergy, suggesting that the introduction of a sufficient amount of such foods in the time window of 3–6 months might prevent the allergy. Unfortunately, the 69.1% drop-out rate of infants recruited in EIG represented a source of bias in the PP analysis [26]. Analyzing the protocol adherence rate for each food, the lowest value was experienced for egg (43.1%), suggesting that the intake of cooked egg may be the key reason for the drop-out. It is noteworthy that the development of food protein-induced enterocolitis syndrome (FPIES) in the EIG was more common with respect to egg than the other foods (six egg, one sesame). In the SIG, three infants developed FPIES (one fish and prawn, one milk and one milk, soya and rice). The difference between the two groups was not significant (*p* = 0.34). However, in the EIG, FPIES frequency and number of cases caused by egg were higher than predictable [26]. These findings might suggest a causative role of the early introduction of egg [25,27].

The Preventing Atopic Dermatitis and Allergies in Children (PreventADALL) trial examines whether the introduction of four allergenic foods at 3–4 months and/or the use of oil-baths can prevent the development of FA in the general population [28]. We hope that the results of this trial are reported soon.

## 3. Further Trials on Peanut

### Introduction from 4–11 Months

The Learning Early About Peanut Allergy (LEAP) [29] (Table 1) trial assessed the effectiveness of early introduction versus the avoidance of peanuts in 640 infants at risk of FA with moderate–severe eczema and/or egg allergy. Parents were instructed to administer at least 6 g of peanut protein contained with Bamba or peanut butter every week, divided in three or more meals per week. At five years of age, children randomized to early introduction and regular ingestion of peanut developed significantly less peanut allergy than those who avoided it (3% vs. 17%). Peanut-specific IgG and IgG4 levels had a significantly greater increase over time in the peanut consumption group. LEAP-On, the follow-up trial, found persistence of the significant decrease of peanut allergy after one year of discontinuing peanut ingestion among infants who had early introduction, as compared to those who had avoided peanuts [30]. The authors used the term “early introduction”, although the median age of participants was 7.8 months with a range of 4–11 months [29], in contrast to a delayed introduction (after two years of age), as previously indicated in international guidelines [1,2,3,8]. However, this preventive effect allows us to assume that the time window to develop immune tolerance is not limited to the period of 4–6 months of age, but may extend up to 11 months, making it reasonable that the immune system may follow a progressive maturation [31,32].

The LEAP trial only enrolled infants younger than 11 months of age. Another study, Preventing Peanut Allergy in Atopic Dermatitis (PEAAD), is a currently ongoing trial that enrolls eczematous children aged 5–30 months. Participants avoid or consume peanuts for one year, following which, peanut allergy is assessed. The PEEAD trial will be able to provide data on the effect of peanut ingestion or avoidance on the development of peanut allergy, after the first year of life, in children at high risk [33].

## 4. Further Trials on Egg

Randomized placebo-controlled trials showed conflicting results concerning the preventive effect of early egg introduction on clinical hypersensitivity reactions to egg (Table 1).

### 4.1. Introduction from 4–6 Months

The Solid Timing for Allergy Research (STAR) trial [34] investigated whether daily doses of pasteurized whole egg, in comparison with a placebo (rice powder), prevented the development of egg allergy in four-month-old infants with moderate–severe atopic eczema, who had never eaten egg. This study was stopped early for ethical reasons: a high frequency of allergic reactions to pasteurized egg (31%), including one case of anaphylaxis, was reported. At 12 months, children with egg allergy were not significantly less in the treated group than in the control group (33% vs. 51%). The rate of egg sensitization was similar in the two groups. Another trial, Hen’s Egg Allergy Prevention (HEAP) [35], examined 383 (of the 406 selected) infants aged 4–6 months from a general population not sensitized to egg. Infants were randomized to receive freeze-dried white egg or placebo three times a week, until one year of age. At 12 months, only 12 infants developed IgE to egg—eight (5.6%) in the active group and four (2.6%) in the placebo group—and egg allergy was found in 2.1% in the active group and 0.6% in the placebo group. Overall, this study failed to find evidence that early egg intake prevents FA and egg sensitization.

Comparable results emerged from the Australian Study Starting Time of Egg Protein (STEP) trial [36] that recruited 820 infants with atopic mothers who had no allergic symptoms and had not previously ingested egg. Infants were daily fed 0.9 g pasteurized raw egg (½ an egg per week) or placebo from 4–6 months to 10 months of age. ITT analysis highlighted no difference between the two groups in the onset of IgE-mediated egg allergy, confirmed by the oral egg challenge (7% active vs. 10.3% placebo) and in cutaneous sensitization (10.8% vs. 15.1%) at 12 months.

The Beating Egg Allergy Trial (BEAT) [37] evaluated high-risk children (at least a relative of first degree affected by atopic disease and skin prick test (SPT) for egg white with diameter <2 mm) who were introduced to pasteurized egg from 4–8 months of age. At 12 months, in the active group, the infants had a significantly lower prevalence of IgE sensitization to egg white than in the placebo group (10.7% vs. 20.5%) and a significant increase of IgG4 levels and IgG4/IgE to egg proteins (no increase in the placebo group). However, no difference was found in the positive egg challenge results between the active group and the placebo group (10.5% vs. 6.2%).

### 4.2. Introduction after Six Months

Different results were found in the Prevention of Egg allergy with Tiny Amount Intake (PETIT) trial [38], which evaluated the efficacy and safety of the introduction of heated egg to prevent egg allergy in 147 high-risk children with atopic eczema, no immediate allergic reaction to egg and no non-immediate reaction to any type of food (Table 1). The infants were randomized to be fed heated egg powder (50 mg/day from 6–9 months and 250 mg/day from 9–12 months) or squash (placebo). The primary outcome (egg allergy confirmed by the open egg challenge at 12 months of age) was not established in 26/147 (17%) infants. The primary analysis included 60 (50%) infants in the egg group and 61 (50%) in the placebo group. At 12 months of age, clinical hypersensitivity reactions to egg were significantly less common in the active group, in comparison to the control group (8% vs. 38%). In addition, at 12 months, levels of IgE to egg white and ovalbumin (OVA) were significantly higher in the placebo group, while IgG4 levels to OVA were significantly increased in the active group. However, at baseline, egg white-specific IgE levels were higher in controls than in the active group. Moreover, the data should be considered with caution, because ITT analysis was not conducted. The primary outcome (egg allergy confirmed by open food challenge at 12 months of age) was not established in 26 (17%) randomized infants

## 5. Does Allergy Come Before Weaning?

Findings of previous studies [39,40] showing that food sensitization can occur before weaning have been reproduced by recent trials for primary prevention of FA. In the STEP trial [36], 5% (18 of 357) of infants who were not previously exposed to egg were sensitized to it (egg-specific IgE (sIgE) > 0.35 kU/L) at 4–6.5 months. In the STAR trial [34], 36% of infants at four months of age with eczema showed positive IgE to egg never ingested before, and 20% (10/49) reacted at the first known exposure. In the HEAP study [35], 5.7% (23/406) of infants who had never previously ingested egg had egg sIgE > 0.35 kU/L and 16 out of 17 children failed the oral challenge for egg. Allergic reactions at first intake of raw pasteurized egg occurred in 4.7% of infants at high risk of atopy and SPT for egg white with diameter <2 mm [34].

With regard to peanuts, in the LEAP study [29], infants who had never been fed peanuts had positive oral peanut challenge at recruitment (7/640), both in the case of positive SPTs (12.8%) and negative SPTs (0.4%). Moreover, children with severe eczema were more likely to have positive SPT or sIgE for any food, even having never ingested it [29]. In the EAT study [25], 5.1% (33/652) of infants in the EIG had positive SPTs to foods never previously eaten at enrollment. Twenty-nine participants underwent challenges for one or more foods, resulting in positive SPT. Oral challenges failed in 3 cases for egg, 4 cases for milk, 2 cases for peanut and 1 case for wheat.

Overall, these findings suggest that other factors, including genetic features, epigenetic modifications and alterations of intestinal flora, may play a pathogenic role in the development of food allergy before weaning. Genetic factors are crucial for the development of FA. However, in human evolution, the increase of FA occurred in a relatively short period of time. This may suggest that it cannot be solely the result of germline genetic mutations . Environmental factors, too, could induce epigenetic changes in gene expression, interrupting the state of food tolerance [41]. In particular, methylation of DNA (DNAm), an epigenetic mechanism that regulates gene expression, seems to play a key role in the differentiation of T cells and in maintaining the Th1/Th2 balance [42,43]. Hong and colleagues found that alterations of DNAm in specific related gene loci at the Th2 response were associated with cow’s milk protein allergy (CMPA) [44]. However, DNAm is a reversible mechanism and can change during the course of the disease. In patients with IgE-mediated CMPA in the active phase, the DNAm of IL-4 and IL-5 (cytokines associated with the Th2 response) was significantly lower, while the DNAm of IL-10 and INF-γ, associated with the Th1 response, was considerably greater than in controls [45]. Moreover, alterations in the composition of commensal bacteria flora during the window of time from the neonatal period to weaning have been associated with atopy development in several mouse models. Germ-free mice, born and raised in a sterile environment, had an exaggerated Th2 immune response, with high levels of IgE, and were more susceptible to anaphylaxis induced by foods, compared to mice colonized with a different microbiota. A possible explanation is that immunomodulatory signals arising from the microbiota are necessary to maintain IgE at baseline levels [46,47,48]. A prospective analysis of a large birth cohort supports the concept that intestinal dysbiosis during the first 100 days of life can influence the development of allergic diseases [49]. Along this line, research has revealed that children born by caesarean section have a higher risk of developing allergic diseases. This may be linked to abnormal colonization of the gut [50]. The lack of exposure to vaginal and perianal bacteria seems to lead to sensitization and the development of FA [51,52].

## 6. From Evidence to Recommendations

There is no clear evidence that the timing of food introduction can reduce the prevalence of clinical hypersensitivity reactions to the same food, with the exception of peanut in infants aged 4–11 months with severe eczema or egg allergy [29] and egg in six-month-old infants with atopic dermatitis [38]. Accordingly, besides the advice to introduce complementary foods, including allergenic foods, any time after four months both in infants at high-risk for atopy and not at high risk, the most recent guidelines, consensus and guidance [53,54,55,56,57] have inconsistently replaced recommendations on the introduction of peanut and egg (Table 2). The consumption of peanuts is directly related to the incidence of peanut allergy, and it greatly varies between countries and regions in the same country. In countries with a high peanut consumption (for example, North America; some countries in South America (Brazil); some European countries, especially the United Kingdom, the Netherlands and Sweden; South-East Asia and India [58]), American guidelines [50] recommend the early introduction of peanut at 4–6 months in infants with severe eczema and/or egg allergy, even if no convincing evidence was found from the LEAP study [28], and at around six months in infants with mild–moderate eczema. The method of peanut introduction depends on the results of IgE or SPT to the food (Table 2). British guidance [57] is not completely in agreement with these recommendations (Table 2). In countries with a low peanut consumption (for example, Colombia, Scandinavia, East and Southern Europe, Australia and Japan [58,59]), guidelines recommend the introduction of peanut according to existing dietary practices. Furthermore, Australian guidelines [56] recommend introducing peanut before 12 months of age. It has also been advised to start around six months, but not before four months. No separate advice for low- and high-risk infants is provided. Along this line, in infants with severe eczema, the Asian Pacific Association of Pediatric Allergy, Respirology and Immunology committee [55] recommend introducing peanut at six months of age. The methods of peanut introduction depend on the availability of IgE tests (Table 2). Lastly, the European suggestion [54] is that peanut introduction should start between four and 11 months in high-risk infants (severe eczema, egg allergy or both). The dissimilarity in recommendations may be due to differences in peanut allergy epidemiology and peanut consumption between countries, as well as the heterogeneity of study designs among published randomized trials. Further studies are, therefore, warranted to clarify whether earlier peanut introduction may increase or prevent peanut allergy in countries with low peanut consumption.

Regarding egg, a meta-analysis [60] selected five studies [34,35,36,37,38] and found moderate-certainty evidence that egg introduction at 4–6 months reduces the development of egg allergy. However, four trials [34,35,36,37] in which raw egg was introduced before six months did not show any protective effect, while the introduction of heated egg beyond six months of age reduced egg allergy [38]. Thus, existing data may only support the introduction of cooked egg at 6–8 months of age [56]. Recommendations for food allergy prevention [55,56] encourage the introduction of cooked egg between six and 12 months of age. Another issue is the prevention of clinical hypersensitivity reactions to peanut or egg at the first known intake. In countries where peanut consumption is high, guidelines [53,55] advise that high-risk infants (severe eczema and/or egg allergy) take peanut-specific IgE tests between 4–11 months before starting regular intake. Furthermore, in infants with severe eczema, it is advisable to carry out egg-specific SPT/sIgE, prior to introducing it into the diet [55]. Egg and peanut should be administered under medical supervision when sIgE and/or SPT results are positive because of the risk of clinical hypersensitivity reactions at exposure. For infants with negative peanut sIgE and/or a peanut SPT wheal of 2 mm or less, peanut should be introduced at home. Caregivers who have concerns should be given the choice of introducing peanut under medical supervision [53]. Egg should be cooked to favor tolerance and reduce the risk of FPIES. The EAT study [25] showed that the early introduction of foods in the general population did not cause serious allergic reactions, when infants with positive SPT to foods underwent a graded oral challenge. Therefore, infants should take an SPT and, if necessary, a food challenge for previously uningested foods to avoid severe adverse reactions at first ingestion. However, we think that unfortunately, in the real-life scenario, there is no healthcare system that can afford to do this. Different laboratory tests, such as atopy patch tests, are not useful in identifying children at risk of clinical hypersensitivity reactions [61,62].

## 7. Conclusions

Studies on FA prevention by the avoidance of allergenic foods have demonstrated that there is no reason to delay their introduction into the diet [8]. On the other hand, there is no evidence that necessitates the intake of allergenic foods before four months of age for the prevention of allergies in the general population and at-risk infants. Hence, we advise that infants at high risk of allergies should be introduced complementary foods according to familial and cultural habits at about six months of age, according to World Health Organization (WHO) recommendations [63]. It has been shown that exposure before four months of age to allergenic foods is ineffective at preventing allergies [25]. Solid foods should be introduced in accordance with familial and cultural habits and tastes of infants, and when possible, while continuing breastfeeding up to two years of age or beyond [56,63]. In practice, during the first year of life, the child can progressively be introduced to all foods, according to his/her requirements and the ability to chew, keep head still and sit propped up. Allergenic foods that may be inhaled (i.e., nuts) because of immature oral motor skills, should be avoided or given in a form that is safe for infants, such as peanut butter. In infants with FA and/or severe eczema [38] with positive SPT to a specific food, an oral food challenge under medical supervision, before introducing the food into the diet, should be considered. This should be taken into account not only for egg and peanut, but also for the other foods, including cow’s milk, tree nuts and seeds, which contain allergens resistant to heat, pH and enzymatic digestion and capable of sensitization via the oral route. Further studies are warranted to clarify the optimal timing for introducing each food and whether component-resolved diagnosis [64] may help in identifying infants who benefit from the early introduction of food allergens.

## Figures and Tables

**Table 1:** Randomized trials on solid food introduction and development of food allergy. SPT, skin prick test.

Author, Year, Trial, Country	Study	Participants			Intervention			Food Challenge/Age (Years)
		Risk for Atopy	Inclusion Criteria	Active/Control (n)	Type of Food	Proteins	Length of Intake	
Perkin, 2014, EAT, U.K. [25]	RCT	General population	Term infant, breastfeeding ≥3 months	652/651	Milk, peanuts, egg, sesame, fish, wheat	4 g/week	3→6 months	Open or controlled to the 6 intervention foods/1–3
Du Toit, 2015, LEAP, U.K. [29]	RCT	High	Moderate-severe eczema and/or egg-peanut allergy, SPT < 4 mm	319/321	Peanuts (snack/butter)	6 g/week, ≥3 times	4–11 months→5 years	Open or DBPCFC to peanuts/5–6
Palmer, 2013, STAR, Australia [34]	RDBPCT	High	Moderate-severe eczema (SCORAD ≥ 15)	49/37	Egg (whole pasteurized)	0.9 g	4–6→8 months	Open to egg/1
Bellach, 2017, HEAP, Germany [35]	RDBPCT	General population	Egg sIgE < 0.35 kU/L	184/199	Egg (white pasteurized)	2.5 g. 3 times/week	4–6→12 months	Open or DBPCFC to egg/1
Palmer, 2017 STEP, Australia [36]	RDBPCT	High	Atopic mother, no previous egg intake, no eczema	165/154	Egg (white pasteurized)	0.4 g/day	4–6→8 months	Open to egg/1
Tan, 2017 BEAT, Australia [37]	RDBPCT	High	Allergic 1st degree relative, SPT egg white <2 mm	407/413	Egg (whole pasteurized)	0.9 g	4–6→10 months	Open to egg/1
Natsume, 2017, PETIT, Japan [38]	RDBPCT	High	Eczema, never taken egg, no immediate egg allergy	60/61	Egg (cooked lyophilized)	−50 mg/day −250 mg/day	−6→9 months −9→12 months	Open to egg/1

EAT, Enquiring About Tolerance; LEAP, Learning Early About Peanut Allergy; STAR, Solids Timing for Allergy Research; HEAP, Hen’s Egg Allergy Prevention; STEP, Starting Time of Egg Protein; PETIT, Prevention of Egg allergy with Tiny Amount Intake; BEAT, Beating Egg Allergy Trial; RDBPCT: Randomized Double-Blind, Placebo-Controlled Trial; RCT: Randomized Controlled Trial; DBPCFC: Double-Blind, Placebo-Controlled Food Challenge; sIgE, specific Immunoglobulin E.

**Table 2:** Recent recommendations for food introduction to prevent food allergy in the general population and in infants at high risk.

Scientific Society–Year	Recommendations
Australasian Society of Clinical Immunology and Allergy (ASCIA), 2016 [56]	- Around 6 months, but not before 4 months. - Peanut and cooked egg before 12 months. - In infants with severe eczema or egg allergy or other food allergies, it should be discussed with a doctor how to do introduction of peanuts.
National Institute of Allergy and Infectious Diseases (NIAID), 2017 [53] §	**Infants without eczema or food allergy** - Peanuts should be introduced into the diet according to the age and to the preferences and cultural habits of the family
	**Infants with mild–moderate eczema** - Introduction of peanuts around 6 months of age, in accordance with family habits.
	**Infants with severe eczema and/or egg allergy** - Introduction of peanuts at 4–6 months after performing sIgE or SPT to peanut. - Infants with peanut sIgE < 0.35 kUA/L and/or peanut SPT wheal of 2 mm or less, should introduce peanut at home or in the office when there are parental concerns. - Infants with peanut sIgE > 0.35 kUA/L and/or peanut SPT wheal of 3–7 mm should perform supervised oral peanut challenge. - Infants with peanut SPT wheal >8 mm are probably allergic to peanuts. They should continue to be managed by a specialist.
European Society for Paediatric Gastroenterology, Hepatology, and Nutrition (ESPGHAN), 2017 [54] §	- Traditions and feeding patterns in the population on types of complementary foods should be considered. - Complementary foods ≥4–6 months. - Allergenic foods ≥4 months. - Infants at high risk of peanut allergy (severe eczema, egg allergy or both) should introduce peanut between 4 and 11 months; following evaluation by an appropriately-trained professional.
Asian Pacific Association of Pediatric Allergy, Respirology and Immunology (APAPARI), 2018 [55]	**General population/at-risk infants (atopic family history, non-severe eczema)** - Complementary foods (including allergenic foods) ≥6 months. - Continue breastfeeding up to 2 years.
	**High risk infants with severe eczema** - Allergy testing (skin prick tests and/or sIgE to egg) (+ peanut in countries with high peanut allergy prevalence) should be preliminary performed followed by a supervised oral challenge in sensitized children. - In countries with limited access to allergy tests. - Expertise-only supervised oral challenges to egg (+ peanut in countries with high peanut allergy prevalence) should be performed. - Introduction of allergenic foods should not be delayed.
British Society for Allergy and Clinical Immunology/British Dietetic Association, 2018 [57]	**General population** - Complementary foods (including allergenic foods) from around 6 months.
	**High risk infants with eczema (particularly early-onset or moderate–severe eczema) or food allergy** - Introduction of egg and peanut from 4 months. - The benefits of allergy testing prior to introducing egg and peanut should be balanced against the risk of a delayed introduction.

§ Recommendations according to the Grading of Recommendations Assessment, Development and Evaluation (GRADE).
